# Development of Randomized Trials in Adults with Medulloblastoma—The Example of EORTC 1634-BTG/NOA-23

**DOI:** 10.3390/cancers13143451

**Published:** 2021-07-09

**Authors:** Peter Hau, Didier Frappaz, Elizabeth Hovey, Martin G. McCabe, Kristian W. Pajtler, Benedikt Wiestler, Clemens Seidel, Stephanie E. Combs, Linda Dirven, Martin Klein, Antoinette Anazodo, Elke Hattingen, Silvia Hofer, Stefan M. Pfister, Claus Zimmer, Rolf-Dieter Kortmann, Marie-Pierre Sunyach, Ronan Tanguy, Rachel Effeney, Andreas von Deimling, Felix Sahm, Stefan Rutkowski, Anna S. Berghoff, Enrico Franceschi, Estela Pineda, Dagmar Beier, Ellen Peeters, Thierry Gorlia, Maureen Vanlancker, Jacoline E. C. Bromberg, Julien Gautier, David S. Ziegler, Matthias Preusser, Wolfgang Wick, Michael Weller

**Affiliations:** 1 Wilhelm Sander-NeuroOncology Unit, Regensburg University Hospital, 93053 Regensburg, Germany; 2 Department of Neurology, Regensburg University Hospital, 93053 Regensburg, Germany; 3 Neuro-Oncology Unit, Centre Léon Bérard, 69008 Lyon, France; Didier.Frappaz@ihope.fr; 4 Department of Medical Oncology, Sydney 2052, Australia; Elizabeth.Hovey@health.nsw.gov.au; 5 Nelune Comprehensive Cancer Centre, Prince of Wales Cancer Centre, Sydney 2031, Australia; Antoinette.Anazodo@health.nsw.gov.au; 6 Faculty of Medicine, Biology and Health, University of Manchester, Manchester Academic Health Science Centre, Manchester M20 4GJ, UK; Martin.McCabe@manchester.ac.uk; 7 Hopp-Children’s Cancer Center Heidelberg (KiTZ), Division of Pediatric Neurooncology, German Cancer Research Center (DKFZ), 69120 Heidelberg, Germany; K.Pajtler@kitz-heidelberg.de (K.W.P.); S.Pfister@kitz-heidelberg.de (S.M.P.); 8 Department of Pediatric Hematology and Oncology, Heidelberg University Hospital, 69120 Heidelberg, Germany; 9 Department of Diagnostic and Interventional Neuroradiology, Klinikum Rechts der Isar der Technischen Universität München, TUM School of Medicine, 81675 Munich, Germany; B.Wiestler@tum.de (B.W.); Claus.Zimmer@tum.de (C.Z.); 10 Department of Radiation-Oncology, University Hospital Leipzig, 04103 Leipzig, Germany; Clemens.Seidel@medizin.uni-leipzig.de (C.S.); Rolf-Dieter.Kortmann@medizin.uni-leipzig.de (R.-D.K.); 11 Department of Radiation Oncology, Klinikum Rechts der Isar der Technischen Universität München, TUM School of Medicine, 81675 Munich, Germany; Stephanie.Combs@tum.de; 12 Department of Neurology, Leiden University Medical Center, 2300 RC Leiden, The Netherlands; L.Dirven@lumc.nl; 13 Department of Neurology, Haaglanden Medical Center, 2501 CK The Hague, The Netherlands; 14 Department of Medical Psychology, Amsterdam UMC, Vrije Universiteit Amsterdam, 1081 HV Amsterdam, The Netherlands; M.Klein@amsterdamumc.nl; 15 Brain Tumor Center Amsterdam at Amsterdam UMC, Vrije Universiteit Amsterdam, 1081 HV Amsterdam, The Netherlands; 16 Kids Cancer Centre, Sydney Children’s Hospital, Sydney 2031, Australia; D.Ziegler@unsw.edu.au; 17 School of Women’s and Children’s Health, University of New South Wales, Sydney 2031, Australia; 18 Department of Neuroradiology, University Hospital Frankfurt, Goethe University, 60528 Frankfurt, Germany; Elke.Hattingen@kgu.de; 19 Department of Neurology, University Hospital Zurich, 8091 Zurich, Switzerland; silvia.hofer@usz.ch (S.H.); Michael.Weller@usz.ch (M.W.); 20 Department of Radiation Oncology, Centre Leon Berard, 69008 Lyon, France; Marie-Pierre.Sunyach@lyon.unicancer.fr (M.-P.S.); Ronan.Tanguy@lyon.unicancer.fr (R.T.); 21 Department of Radiation Oncology, Royal Brisbane and Women’s Hospital, Brisbane 4029, Australia; Rachel.Effeney@health.qld.gov.au; 22 Department of Neuropathology, University Hospital Heidelberg, 69120 Heidelberg, Germany; Andreas.vonDeimling@med.uni-heidelberg.de (A.v.D.); Felix.Sahm@med.uni-heidelberg.de (F.S.); 23 Clinical Cooperation Unit Neuropathology, German Consortium for Translational Cancer Research (DKTK), German Cancer Research, 69120 Heidelberg, Germany; 24 Department of Pediatric Hematology and Oncology, University Medical Center Hamburg-Eppendorf, 20246 Hamburg, Germany; S.Rutkowski@uke.de; 25 Division of Oncology, Department of Medicine I, Medical University of Vienna, 1090 Vienna, Austria; Anna.Berghoff@meduniwien.ac.at (A.S.B.); Matthias.Preusser@meduniwien.ac.at (M.P.); 26 Medical Oncology Department, Azienda USL/IRCCS Institute of Neurological Sciences, 40139 Bologna, Italy; Enricofra@yahoo.it; 27 Barcelona Translational Genomics and Targeted Therapeutics in Solid Tumors Group, Department of Medical Oncology, Hospital Clinic Barcelona, 08036 Barcelona, Spain; Epineda@clinic.cat; 28 Department of Neurology, Odense University Hospital, DK-5000 Odense, Denmark; Dagmar.beier@rsyd.dk; 29 EORTC Headquarters, 1200 Brussels, Belgium; Ellen.Peeters@eortc.be (E.P.); Thierry.Gorlia@eortc.be (T.G.); Maureen.Vanlancker@eortc.be (M.V.); 30 Erasmus Medical Center Cancer Institute, Department of Neuro-Oncology, 3015 GD Rotterdam, The Netherlands; j.bromberg@erasmusmc.nl; 31 Clinical Research Department, Centre Léon Bérard, 69008 Lyon, France; Julien.Gautier@lyon.unicancer.fr; 32 Children’s Cancer Institute, University of New South Wales, Sydney 2031, Australia; 33 Department of Neurology, University Hospital Heidelberg, 69120 Heidelberg, Germany; Wolfgang.Wick@med.uni-heidelberg.de; 34 Clinical Cooperation Unit Neuro-Oncology, German Consortium for Translational Cancer Research (DKTK), German Cancer Research, 69120 Heidelberg, Germany

**Keywords:** medulloblastoma, adult, clinical trial, randomized, subgrouping, cerebrospinal fluid, magnetic resonance imaging, radiotherapy, chemotherapy, targeted therapy

## Abstract

**Simple Summary:**

Medulloblastoma is rare after puberty. Among several molecular subgroups that have been described, the sonic hedgehog (SHH) subgroup is highly overrepresented in the post-pubertal population and can be targeted with smoothened (SMO) inhibitors. However, no practice-changing prospective clinical trials have been published in adults to date. Tumors often recur, and treatment toxicity is relevant. Thus, the EORTC 1634-BTG/NOA-23 trial for post-pubertal patients with standard risk medulloblastoma will aim to increase treatment efficacy and to decrease treatment toxicity. Patients will be randomized between standard-dose vs. reduced-dosed radiotherapy, and SHH-subgroup patients will also be randomized between the SMO inhibitor sonidegib (Odomzo^TM,^, Sun Pharmaceuticals Industries, Inc., New York, USA) in addition to standard radio-chemotherapy vs. standard radio-chemotherapy alone. In ancillary studies, we will investigate tumor tissue, blood and cerebrospinal fluid samples, magnetic resonance images, and radiotherapy plans to gain information that may improve future treatment. Patients will also be monitored long-term for late side effects of therapy, health-related quality of life, cognitive function, social and professional live outcomes, and reproduction and fertility. In summary, EORTC 1634-BTG/NOA-23 is a unique multi-national effort that will help to council patients and clinical scientists for the appropriate design of treatments and future clinical trials for post-pubertal patients with medulloblastoma.

**Abstract:**

Medulloblastoma is a rare brain malignancy. Patients after puberty are rare and bear an intermediate prognosis. Standard treatment consists of maximal resection plus radio-chemotherapy. Treatment toxicity is high and produces disabling long-term side effects. The sonic hedgehog (SHH) subgroup is highly overrepresented in the post-pubertal and adult population and can be targeted by smoothened (SMO) inhibitors. No practice-changing prospective randomized data have been generated in adults. The EORTC 1634-BTG/NOA-23 trial will randomize patients between standard-dose vs. reduced-dosed craniospinal radiotherapy and SHH-subgroup patients between the SMO inhibitor sonidegib (Odomzo^TM^, Sun Pharmaceuticals Industries, Inc., New York, USA) in addition to standard radio-chemotherapy vs. standard radio-chemotherapy alone to improve outcomes in view of decreased radiotherapy-related toxicity and increased efficacy. We will further investigate tumor tissue, blood, and cerebrospinal fluid as well as magnetic resonance imaging and radiotherapy plans to generate information that helps to further improve treatment outcomes. Given that treatment side effects typically occur late, long-term follow-up will monitor classic side effects of therapy, but also health-related quality of life, cognition, social and professional outcome, and reproduction and fertility. In summary, we will generate unprecedented data that will be translated into treatment changes in post-pubertal patients with medulloblastoma and will help to design future clinical trials.

## 1. Introduction

Medulloblastoma is a rare condition in adult neuro-oncology practice [1]. Around 70% of cases occur in patients under 15 years of age, and the peak incidence is around 5 years of age [2]. The US registry analysis from the Surveillance, Epidemiology, and End Results (SEER) database reported that the incidence of medulloblastoma was 0.6 cases per million in adults [3]. One can assume that the incidence in Europe is similar to the US, summing up to approximately 450 newly diagnosed patients per year in Europe. In post-pubertal patients, medulloblastoma is equally distributed between sexes [1,4].

A staging system was introduced by Chang et al. in 1969 to describe the extent of tumoral infiltration (T1–T4) and metastases (M0-M4) in medulloblastoma [5]. T-stage likely has a prognostic role in adults [6,7]. Concerning metastasis, M1-M4 is considered high-risk in pediatric patients [8,9]. Whether M1 disease has prognostic value in post-pubertal patients and adults is, however, still a matter of debate.

Depending on genetic subgroups, adult and pediatric medulloblastomas are distinct [10,11,12], which mandates the development of molecularly adapted treatment strategies. In addition to molecular differences, clinically relevant features clearly differentiate adult from pediatric medulloblastoma. For example, adolescents and adults have a higher incidence of lateral localization of the tumor in the hemispheres of the cerebellum than children [13,14]. This localization relates to the known overrepresentation of the SHH subgroup in adults [10]. In addition, and in contrast to children, SHH mutations in adults are at the level of SMO or upstream in the vast majority [15]. While treatment has evolved, based on the results of successive randomized clinical trials, in a step-wise fashion in children, it has never been optimized for post-pubertal patients in a prospective, randomized way [16]. Treatment has therefore been extrapolated from experiences in children, and personalized or molecularly stratified therapies have not been developed in adults. The most common treatment regimen in adults is radiotherapy of the craniospinal axis only or radiotherapy combined with the Packer [17] or Taylor [18] chemotherapy regimen at this time. Furthermore, considering the current associated widespread variation in treatment algorithms both within and between countries, multi-national prospective randomized trials in the post-pubertal and adult population are highly warranted. Such trials will be instrumental in improving treatment guidelines and counseling for the design of future clinical trials in this population [16]. In addition, treatment toxicity is high and often includes declines in neurological function, hearing, and cognition, connected to severe impairments in quality of life and social and professional function [19,20,21,22,23], which also mandates approaches to decrease the detrimental side effects of treatment.

Efficacy data for combined radio-chemotherapy for adult patients with medulloblastoma are scarce. The Packer chemotherapy regimen [17,24] has set the basis for a series of pediatric trials [23,25,26] and has also been used in adults. It consists of craniospinal radiotherapy plus weekly vincristine 1.5 mg/m^2^ (maximum 2 mg), followed by a maximum of eight cycles of lomustine 75 mg/m^2^ and cisplatin 70 mg/m^2^ on day 1 and vincristine 1.5 mg/m^2^ (max. 2 mg) on day 1, 8, and 15 of six weekly cycles. In a retrospectively evaluated cohort of the pediatric HIT-2000 trial, 49 non-metastatic adults who received combined radio-chemotherapy experienced a 4-year event-free survival of 74% and OS of 94% [23]. These and other data constitute the basis for a compiled estimated 3-year progression free-survival in adults of around 73% [7,21,23,27]. Alternative chemotherapy regimens that move away from a purely Packer-based adjuvant chemotherapy in favor of a less toxic alternating cisplatin/cyclophosphamide regimen have not yet been tested in adults.

## 2. Classification of Medulloblastoma

According to the concept of an integrated diagnosis of the current and upcoming WHO classifications of tumors of the CNS, medulloblastoma subgroups must be defined by both histological and molecular/genetic features [28]. All medulloblastoma subgroups correspond to WHO grade 4. The WHO classification of 2016, as well as the upcoming 2021 classification [28], describes five molecularly defined medulloblastoma subgroups—WNT-activated, SHH-activated/TP53 mutated, SHH-activated/TP53 wild-type, group 3, and group 4—and four histological patterns—classic, desmoplastic/nodular, with extensive nodularity, and large cell/anaplastic. There is no perfect concordance of the genetic and histological subgroups at this time.

Diagnosis can be further refined by DNA sequencing, which reveals exact mutations, with the advantage of informing a clinician regarding the potential suitability of a patient for consideration of targeted therapies in SHH medulloblastoma. In adults, SHH-activated, TP53 wild-type medulloblastomas represent the most frequent subgroup, with around 60 to 70% of cases [11]. SHH activation is typically caused by mutations of *PTCH1* or *SMO* in most adult cases, coding for central critical cell membrane-associated components of the SHH pathway that are potentially actionable with SMO inhibitors [15]. SHH tumors have similar outcomes in infants (5-year overall survival (OS) rate of 67.3–88.0%), children (5-year OS rate of 69.8%), and adults (5-year OS rate of 88.5%) [12], with differential—and sometimes worse—outcomes for the adult subgroup in the older literature. This is likely associated with a specific biology that leads to desmoplastic histology among SHH tumors in infants. Subgroups of patients with certain germline alterations such as TP53, BRCA2, and PALB2 [29] as well as amplifications of MYC/MYCN [30] bear a worse prognosis. However, germline mutations are less commonly found in adults in comparison to children, and most mutations are somatic in this population [31].

## 3. Backbone of Therapeutic Strategy in EORTC 1634-BTG/NOA-23

### 3.1. Resection

Most patients initially present with hydrocephalus or symptoms from mass effect caused by the tumor. A gross total resection of the primary tumor should therefore be considered in all patients to alleviate symptoms and to facilitate rapid diagnosis [32]. In patients with group 4 tumors, there is an established association between improved progression-free survival and gross total resection. In cases where gross total resection is not safe and/or feasible, a maximal safe resection, sparing eloquent areas and leaving residual tumor behind, should be performed [33]. Best possible resection will therefore be a mainstay of EORTC 1634-BTG/NOA-23 to set the stage for an effective therapy and also to provide sufficient tissue for histological and molecular diagnosis and subgrouping.

### 3.2. Radiotherapy

Radiotherapy is an essential component of the combined modality treatment of medulloblastoma and also the backbone of EORTC 1634-BTG. Given the propensity of medulloblastoma to disseminate via the CSF, EORTC 1634-BTG will follow the current practice in non-infants with confirmed medulloblastoma that consists of craniospinal irradiation (CSI) with a boost to the tumor bed [34].

In pediatric cohorts, the quality of radiotherapy strongly relates to survival and functional outcome. Several reports showed that inadequate treatment had a negative impact on tumor control and survival [35,36]. A more recent analysis of the PNET5 trial underlined the necessity of pretreatment central quality control [37]. Radiotherapy should be initiated within 4–6 weeks after surgery, and the course of radiotherapy should ideally be performed without interruptions [37,38].

Historically, CSI is delivered with a total dose of 36 Gy in daily fractions of 1.8 Gy, or of 35.2 Gy in daily fractions of 1.6 Gy, each five times weekly. In addition, a local dose escalation to the posterior fossa or, more recently, tumor bed [39] is performed as a boost treatment. Generally, a total dose up to 54/55.8 Gy should be achieved in the boost region [6]. Since radiotherapy can generate dose-dependent long-term side effects, dose reduction of craniospinal axis radiotherapy has been evaluated in clinical trials in children. A neuroaxis dose reduction from 36 Gy to 23.4 Gy in combination with chemotherapy has been used in pediatric trials [17] and was equivalent in all published trials. The current PNET5 trial will evaluate if an even lower total CSI dose of 18.0 Gy is possible in good prognostic subgroups. The question of radiotherapy dose reduction has not been investigated in a randomized way in adults; however, single-arm data indicate that a dose reduction to 23.4 Gy is feasible without losing efficacy [40]. A phase III trial for patients up to 21 years of age with standard-risk disease showed that a CSI dose of 23.4 Gy in 13 fractions with local dose escalation to the tumor bed of 32.4 Gy in 18 fractions demonstrated similar efficacy to contemporary studies of higher-dose craniospinal radiotherapy [41].

Radiotherapy-related toxicity is of concern, especially in patients with long-term survival, where chronic side effects become relevant. It may include declines in neurocognitive and neuropsychological functioning, social skills, attention, and reading [42,43,44]. The effect of CSI on cognition is dose-dependent [45,46]. Moreover, radiotherapy of the spinal axis may contribute to a risk of gonadal dysfunction and subsequent fertility issues in female patients caused by scatter irradiation. Long-term survivors of childhood medulloblastoma are also at increased risk of secondary tumors, hearing impairment, stroke, poor balance, and cataracts [47]. Of caution, the risk for certain side effects, particularly hematological side effects, is higher in older patients. Therefore, high-precision radiotherapy techniques to spare the vertebrae represent an important issue [48]. Radiotherapy dose reduction with the aim of reducing radiotherapy toxicity will therefore be the most important clinical secondary endpoint of EORTC 1634-BTG. Based on the referenced data, we will adhere to a 35.2 Gy dose to the craniospinal axis in the standard arm, except for patients below age 18, who will receive 23.4 Gy. All patients with M0 disease in the experimental arm will receive a reduced 23.4 Gy dose to the craniospinal axis. Patients with M1 disease will receive 35.2 Gy.

If available, proton beam therapy can be considered an alternative to helical tomotherapy or volumetric modulated arc therapy (VMAT) to reduce the radiation dose outside of the target volume and therefore reduce the risk of short-term and long-term side effects [49,50]. Similar survival outcomes were reached in children with proton compared to photon irradiation [51,52]. However, there are only a few prospective comparisons between photon and proton treatment with regard to short- and long-term toxicity as well as disease outcomes. Proton beam therapy will be encouraged in EORTC 1634-BTG and its safety and efficacy will be investigated as a secondary endpoint in comparison to photon therapy.

### 3.3. Combined Radio-Chemotherapy

Data from recent meta-analyses [53,54] and prospective single-arm trials [21,23,27,55,56] strongly suggest a beneficial role of chemotherapy in addition to radiotherapy in adult patients with medulloblastoma. A recent meta-analysis by Kocakaya et al. analyzed 227 publications with 907 patients. Patients who received chemotherapy first-line survived significantly longer (median OS, mOS: 108 months, 95% CI: 68.6–148.4) than patients treated with radiotherapy alone (mOS: 57 months, 95% CI: 39.6–74.4) [53]. In a similar meta-analysis, radio-chemotherapy was associated with a superior mOS compared with radiotherapy alone (HR: 0.53; 95% CI: 0.32–0.88, *p* = 0.01) [54].

Prospective single-arm trials in adults and retrospectively evaluated subpopulations from pediatric trials corroborate a survival gain through radio-chemotherapy in comparison to radiotherapy. In a prospective trial by Brandes et al. [55,57], 26 high-risk patients received two cycles of upfront chemotherapy, either with a MOPP-like regimen or with cisplatin, etoposide, and cyclophosphamide, followed by radiotherapy and maintenance chemotherapy. After a median follow-up of 7.6 years, the overall PFS and OS rates at 5 years were 72% and 75%, respectively. A further analysis of this trial with a median follow-up of 10 years showed that low-risk patients who had received cisplatin-based chemotherapy after radiotherapy obtained 5-year and 10-year OS rates of 100%, compared to 5-year and 10-year OS rates of 100% and 78.6% in patients treated with radiotherapy alone [7,27]. The HIT-2000 trial generated an adult observational cohort of 49 patients with non-metastatic disease patients who received combined radio-chemotherapy with eight doses of vincristine 1.5 mg/m^2^ (maximum 2 mg) during radiotherapy, followed by a maximum of eight cycles of lomustine 75 mg/m^2^ day 1, cisplatin 70 mg/m^2^ day 1, and vincristine 1.5 mg/m^2^ (max. 2 mg) on day 1, 8, and 15 of six-weekly cycles and experienced a 4-year event-free survival rate of 74% and overall survival rate of 94% [23]. This regimen has been prospectively evaluated with regard to toxicity aspects within the NOA-07 trial [21]. In NOA-07, toxicity was moderate, with 70% of patients tolerating at least four cycles of chemotherapy, all of them with dose modifications. Feasibility appeared to be age-dependent, leading to the application of four cycles of chemotherapy in 72.7% of patients below age 45 and 62.5% of patients aged 45 or above (*p* = 0.66). Assessing for the specific outcome of completion of all eight maintenance cycles demonstrated that 45.5% of all patients younger than 45 years completed eight cycles, whereas only 12.5% of patients over 45 years received all cycles (*p* = 0.199). Severe adverse events were significantly more frequent in patients older than 45 years of age (*p* = 0.040). No treatment-related deaths were observed. Leukopenia was the major toxicity. Polyneuropathy and ototoxicity were the only grade 3 and 4 non-hematological toxicities [21].

A combined view on the efficacy and tolerable toxicity of all previously used options in the adult setting led to the selection of NOA-07 (HIT-2000, Packer) as the suitable radio-chemotherapy regimen for the standard arm of a prospective trial in post-pubertal and adult patients with medulloblastoma. Based on published data, we concluded that lowering the dosing frequency of vincristine to every second week and the number of maintenance cycles to six would allow clinicians to treat more than 50% of patients with the full regimen and acceptable resultant toxicity. As we intend to adhere as much as possible to published data, we will not eliminate vincristine from the chemotherapy regimen. Additionally, carboplatin might be a pragmatic substitute for cisplatin, based on its more favorable toxicity profile [23,58]. However, no published data thus far have systematically replaced cisplatin by carboplatin in adults, and it is therefore deemed not justified to routinely replace cisplatin with carboplatin in the EORTC 1634-BTG/NOA-23 trial. However, if cisplatin-related side effects occur, investigators will be free to substitute cisplatin by carboplatin. Strict tapering and stopping rules will apply that will allow early and fast de-escalation and discontinuation of the drugs used in EORTC 1634-BTG/NOA-23, if presumptively related symptoms occur.

### 3.4. Targeted Therapy

Medulloblastoma is well understood on a molecular level, and two of the molecular subgroups, SHH and WNT, are driven by pivotal signaling pathways that are, in principle, amenable to targeted therapies [59,60]. A series of clinical trials have been initiated that target specific molecular subgroups of medulloblastoma [61,62].

At this point, the sonic hedgehog (SHH) subgroup is the population of choice for a personalized targeted intervention in adults with medulloblastoma, as this subgroup constitutes the majority of adult patients (60–70%) [11,63], and specific inhibitors of smoothened (SMO), an upstream member of the SHH signaling pathway, are available. In addition, the use of SMO inhibition as a mechanism to reduce SHH pathway activation holds excellent biological rationale, as adult patients within the SHH subgroup have a very low frequency of mutations downstream of SMO [10,15].

Animal model data [64] and non-randomized trials in patients with SHH-driven tumors [65,66] generated impressive results. An activated hedgehog pathway predicts response to SMO inhibition in these tumors [67]. Sonidegib is a potent oral SMO inhibitor, which showed efficacy in patients with solid tumors [68] and was evaluated in a phase II (ClinicalTrials.gov: NCT01125800, 26 May 2020) and a phase III trial (ClinicalTrials.gov: NCT01708174, 26 May 2020) in pediatric and adult patients with medulloblastoma [69]. One of the reasons for low recruitment in these trials was that SMO inhibitors induce premature growth plate fusions in children [70], a side effect that is fortunately not relevant in post-pubertal patients and therefore not limiting when growth plates have already fused. In the EORTC 1634-BTG/NOA-23 study, only post-pubertal and adult patients will be included, avoiding this problem. The recently published MEVITEM trial evaluated vismodegib, another SMO inhibitor, plus temozolomide in immunohistochemically defined, recurrent, SHH-driven adult medulloblastoma. In 10 patients in the combination arm, PFS-6 was 20% and the overall response rate was 40% (95% CI: 12.2; 73.8), and among 11 patients with an expected sensitivity according to next-generation sequencing, three had a partial response and four remained stable. The authors concluded that the prediction of sensitivity to vismodegib needs further refinements [62]. In addition, sensitivity to SMO inhibition is likely lower in this relapsed cohort in comparison to a therapy-naïve cohort due to the genetic divergence between primary and relapsed disease [71]. Of note, sonidegib also seems to be more effective in comparison to vismodegib in patients with medulloblastoma [72].

Data on the pharmacokinetics of sonidegib suggest favorable blood–brain barrier penetration [68,73]. As medulloblastomas are associated with a disrupted blood–brain barrier [74] that correlates to contrast enhancement in MRI [75], sufficient intra-tumoral drug levels can be anticipated. In addition, sonidegib will be given in combination with radiotherapy in EORTC 1634-BTG/NOA-23, which is thought to further disrupt the blood–brain barrier, at least in the short term [76].

Sonidegib has a favorable toxicity profile with rare haematological toxicities in adults, which is an important point if a combination therapy with classical chemotherapeutics and radiotherapy is considered. The most frequently reported adverse events with combined therapy including sonidegib in 230 patients with basal cell carcinoma were muscle spasms (54%), musculoskeletal pain (32%), and myalgia (19%). Increased serum creatin kinase (CK) laboratory values occurred in 61% of patients, with 8% of patients having grade 3 or 4 serum CK elevations [77]. In a pooled safety analysis of 12 clinical studies involving 571 patients with various advanced cancers treated with sonidegib at daily doses ranging from 100 mg to 3000 mg, rhabdomyolysis occurred in one patient (0.2%) treated with sonidegib 800 mg. Other relevant side effects were alopecia, dysgeusia, decreased appetite, nausea, diarrhea, fatigue, abdominal pain, headache, pruritus, and minor effects on bone marrow. Therefore, the treatment-limiting side effects of sonidegib do not overlap with the typical side effects of radiotherapy, besides a possible, but minor, additional toxicity on bone marrow. As CSI and sonidegib have not been investigated in a combined manner yet, a run-in phase will be performed in EORTC 1634-BTG/NOA-23.

## 4. Trial Design

**Figure 1:**
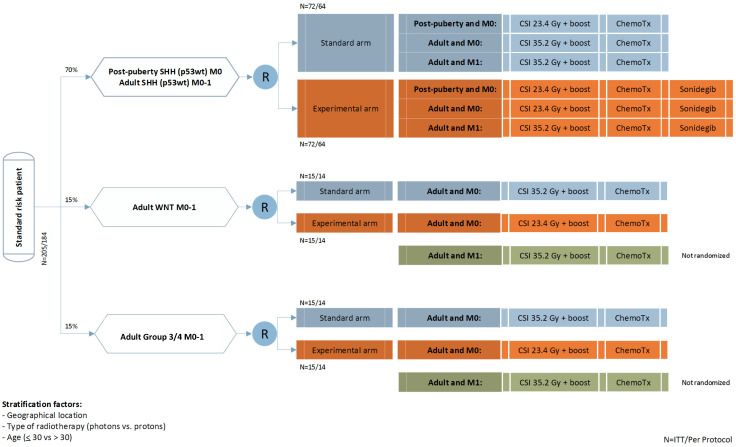
Trial design: Patients will be stratified into their respective genetic subgroups. Randomization will be performed into regular-dose (35.2 Gy) and low-dose (23.4 Gy) radiotherapy for all strata, and radio-chemotherapy vs. reduced radio-chemotherapy plus sonidegib in the SHH subgroup. Chemotherapy consists of 4 doses of vincristine 1.5 mg/m^2^ (maximum 2 mg) during radiotherapy, followed by a maximum of 6 cycles of lomustine 75 mg/m^2^ on day 1, cisplatin 70 mg/m^2^ on day 1, and vincristine 1.5 mg/m^2^ (max. 2 mg) on day 1 and 15 of 6-weekly cycles. The following modification for post-pubertal patients aged 17 and below with WNT and group 3/group 4 will apply: these patients will not be treated in the EORTC 1634-BTG/NOA-23 trial and will be recommended to participate in a suitable pediatric trial. N = number of patients; R = randomization; SHH, WNT, group 3/group 4 are medulloblastoma subgroups; M = metastasis; CSI = craniospinal irradiation; Gy = gray; ChemoTx = chemotherapy, ITT = intent to treat. Please note that group 3 patients were not included in the initial version of the protocol, but will be included in the amended version 3.0 as depicted.

Considering the biological and prognostic diversity of pediatric and adult medulloblastoma [10,11] and the lack of prospective randomized data in adults, there is an unmet medical need to develop efficacious treatment regimens for these patients. This is also true in view of the intermediate to dismal prognosis for post-pubertal and adult medulloblastoma patients, and the considerable toxicity of craniospinal radiotherapy. The EORTC 1634-BTG/NOA-23 trial (ClinicalTrials.gov: NCT04402073, 26 May 2020) is a multicenter, randomized, controlled, open-label, phase II trial open for M0/M1 medulloblastoma. EORTC 1634-BTG/NOA-23 is the first trial worldwide that prospectively enrolls post-pubertal and adult patients with medulloblastoma according to their molecular subgroup in a randomized design, with the aim to lower treatment toxicity and increase efficacy in these patients (Figure 1).

The 1634-BTG/NOA-23 trial therefore aims at developing a personalized, genotype-based, intensity-modulated therapy for post-pubertal and adult patients with newly diagnosed medulloblastoma. It will generate a study population with well-annotated clinical data that will be connected to translational subprojects. The translational subprojects will enable us to evaluate molecular, radiomic, and radiogenomic data as well as data on health-related quality of life (HR-QoL), neurocognitive functioning, and fertility and endocrine events to gain deeper insights into subgrouping, risk stratification of adult disease, therapeutic targets, resistance mechanisms, and the toxicity of these treatments.

Patients of all medulloblastoma molecular subgroups will be included in the trial and treated. Adult patients with M1 disease will be included in 1634-BTG/NOA-23, as there are no clear data that M1 disease bears an inferior prognosis in adults [53,58,78].

A radio-chemotherapy backbone will be used in the standard as well as the experimental arm. In the experimental arm, CSI dose will be reduced [17,24] and an SMO inhibitor, sonidegib, will be added for patients with SHH-activated medulloblastoma, based on its biological rationale and published clinical data [68,69,73]. Based on the referenced data that suggest equal efficacy of 35 Gy vs. 23.4 Gy CSI doses [17,40,41,79], the reduction of toxicity with dose-reduced CSI while maintaining efficacy will be investigated by using 35.2 Gy in the standard vs. 24.6 Gy in the experimental arm of the trial. The EORTC 1634/NOA-23 chemotherapy regimen will consist of four doses of vincristine 1.5 mg/m^2^ (maximum 2 mg) during radiotherapy, followed by a maximum of six cycles of lomustine 75 mg/m^2^ on day 1, cisplatin 70 mg/m^2^ on day 1, and vincristine 1.5 mg/m^2^ (max. 2 mg) on day 1 and 15 of six-weekly cycles. Considering that post-pubertal patients with SHH-subgroup medulloblastomas are the prime target population for SHH inhibitors [15,67], an increase in efficacy in the SHH subgroup will be the primary efficacy endpoint. As no formal combination data of sonidegib with radiotherapy are available at this time, a run-in phase with 10 patients will be included in EORTC 1634-BTG/NOA-23, who will be observed closely for unexpected toxicity.

The EORTC 1634-BTG/NOA-23 trial will be performed in more than 40 sites in Europe and Australia. A list of activated participating sites can be found at https://www.clinicaltrials.gov/ct2/show/NCT04402073, accessed on 26 May 2020.

## 5. Objectives

The primary objective of 1634-BTG/NOA-23 is to compare progression-free survival of a personalized intensity-modulated therapy (experimental arm; sonidegib) vs. standard therapy in the SHH-dependent subgroup. Secondary objectives include the reduction of radiotherapy toxicity with a dose-reduced CSI while maintaining efficacy and additional efficacy objectives. As patient-reported outcomes are highly important in a setting where young patients in the middle of their lives are affected, short- and long-term health-related quality of life (HR-QoL), neurocognitive function, social outcome, and fertility/endocrine function as well as fertility interventions will be related to these data. Even if the primary objective of the trial should remain negative, the secondary objectives will enable us to better understand the individual value of this risk-stratified personalized therapy approach, which not only bears the chance for enhanced efficacy, but also the risk of enhanced toxicity during the entire disease trajectory.

A number of translational research objectives aim to construct measurable parameters that can predict the clinical outcome of post-pubertal patients with medulloblastoma. This includes a more precise classification of subgroups using molecular data and radio-genomic classifiers, as well as the evaluation of molecular characteristics within the SHH subtype that may explain response patterns, testing of the feasibility of circulating tumor DNA (ctDNA) from cell-free cerebro-spinal fluid (CSF) and blood for molecular subgrouping, detection of minimal residual disease, and detection of new molecular targets, pathway modifiers, and resistance mechanisms in the SHH subgroup. We further will evaluate biomarkers from tumor tissue within the SHH subtype that may explain early side effects and predict radiotherapy toxicity and endocrinology and fertility issues. These translational objectives will also add important information to the design of future trials in medulloblastoma.

## 6. Evaluation of Efficacy and Statistics

Verification of inclusion and exclusion criteria will be done centrally and includes a neuropathology review and molecular subgroup analysis, which classifies patients according to their molecular subgroup (SHH, WNT, group 3, and group 4). Patients will be centrally randomized between the standard and experimental arm. A minimization technique will be used for random treatment allocation stratified by country, type of radiotherapy (proton versus photon), and age (≤30 vs. >30) in the SHH subgroup and type of radiotherapy (proton vs. photon) in the WNT, group 3, and group 4 subgroups. SHH-activated patients under age 18 with M1 disease and patients under age 18 in the WNT-, group 3, and group 4 subgroups will not be enrolled, and will be recommended to participate in the HRMB or PNET5/SIOP trials, depending on their risk profile. Adults with M1 disease in the WNT, group 3, and group 4 subgroups will be enrolled, but not randomized, and will be treated with standard radio-chemotherapy.

The total number of patients to be registered is estimated at 205, including 128 SHH M0-1 eligible patients who started their allocated treatment.

Treatment decisions will be based on the adapted response assessment in pediatric neuro-oncology (RAPNO) criteria [75] as assessed by the local investigator. Imaging will be verified by a central review board at the time point of patient inclusion and suspected tumor progression. Response to treatment will be assessed on the basis of an MRI of the brain and spine, CSF cytology, and the neurologic exam.

The evaluation of progression-free survival (PFS) in patients with SHH-subgroup medulloblastoma in comparison of the standard vs. experimental arm will be powered statistically as the primary endpoint. We hypothesize that the addition of sonidegib to the standard treatment will increase the progression-free survival (PFS) rate at 3 years in a statistically significant and clinically meaningful way. A PFS rate at 3 years equal to 86.6% in the experimental arm (HR, Hazard Ratio, equal to 0.456) is considered clinically relevant compared to an expected PFS-3 of 73.0% in the standard arm for SHH-subgroup patients based on cumulative prior literature [7,21,23,27]. We plan to show this difference at one-sided 10% significance (20% two-sided) and with 90% power and assume a cumulative drop-out rate at 3 years of 5% in each treatment arm. Based on these assumptions, 43 PFS events in the standard arm of the SHH subgroup are needed to evaluate the primary endpoint. For the WNT subgroup, group 3, and group 4, differences in PFS between the treatment arms will be assessed in the intent-to-treat patients (ITT) population. Assuming a monthly accrual of 4.26 patients after an activation period of 1 year, 128 eligible patients from the SHH subgroup who started their allocated treatment will be randomized between the standard arm and experimental arm during 36 months and followed-up for 55 months with a total duration of 91 months.

The evaluation of safety in medulloblastoma patients in the SHH, WNT, group 3, and group 4 subgroups in comparison of the standard vs. experimental arm will be performed with descriptive intent only, based on CTCAE criteria.

## 7. Translational Research

**Figure 2:**
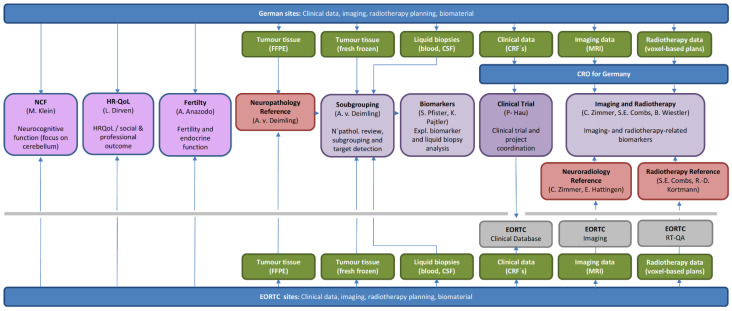
Graphic representation of the overall trial and translational project organization: EORTC is the sponsor of the trial, will host the trial in cooperation with Neuro-Onkologische Arbeitsgemeinschaft in der Deutschen Krebsgesellschaft (NOA), and ensure processing of clinical data, imaging data, and biomaterial that are analyzed within the translational subprojects. Reference sites for neuropathology, neuroimaging, and radiotherapy quality assurance have been defined and will directly receive biomaterial from sites and MRI images and voxel-based radiotherapy plans from the EORTC imaging platform and EORTC quality assurance in radiotherapy (RT-QA) platform. Neuro-cognition (NCF), health-related quality of life (HRQoL), and fertility and endocrine function will be assessed centrally. Responsible sites and principal investigators are named in the respective task boxes. WP = work package; FFPE = formalin-fixed paraffin-embedded; CSF = cerebrospinal fluid; CRF = case report form; MRI = magnetic resonance imaging; N’pathol = neuropathology; CRO = contract research organization.

Several reference and translational work packages are based on the prospective randomized EORTC-1634/NOA-23 phase II trial. The trial backbone will allow the generation of data and bio- as well as imaging material that will be processed to the reference centers and translational work packages (Figure 2).

### 7.1. Neuropathology Reference and Subgrouping

The main aim of the neuropathology reference and molecular grouping part is to provide a state-of-the-art molecular and histological sub-classification of post-pubertal medulloblastoma, to allow for the identification of new molecular targets, pathway modifiers, and resistance mechanisms, as well as to ensure suitable material for further analysis in other work packages [16,60]. The entire trial population will be subjected to central neuropathology assessment, with one European site and one Australian neuropathology site performing these central reviews. After exclusion of non-medulloblastomas, all cases will be molecularly analyzed by methylation-based classification employing the 850 K chip and detection of targetable mutations by next-generation panel sequencing (NGS) [80,81].

### 7.2. Genotype-Based Subgrouping and Target Detection from Liquid Biopsies

In this work package, we aim to identify and establish novel innovative biomarkers for response prediction and treatment monitoring to inform EORTC 1634-BTG/NOA-23 trial interpretation, but also future refined clinical trials. RNA sequencing data from fresh frozen tumor samples generated in this work package will be used for biomarker discovery, including the prediction of treatment response to SMO inhibition, identification of potential resistance mechanisms, and prediction of genotype-associated treatment toxicity. For this purpose, RNA sequencing data will be integrated with DNA methylation array data and panel sequencing data from primary tumor samples generated in the neuropathology subproject as well as circulating tumor DNA (ctDNA) data, imaging, and clinical outcome.

The work package will also test the feasibility of using ctDNA from CSF and blood for molecular subgrouping and treatment monitoring to assess minimal residual disease [82]. CSF and blood will be collected from all patients at baseline. Analyses of ctDNA using NGS gene panel sequencing will be used to track SHH-associated mutations in the CSF and blood. Patients with medulloblastoma predicted as SHH that harbor an SHH-associated mutation will undergo liquid biopsies at three additional time points, following radiotherapy, following chemotherapy, and in case of relapse. The overall ambition is to identify novel, more precise predictive and minimal residual disease biomarkers for response prediction to radiotherapy, conventional chemotherapy, and SMO inhibition in post-pubertal SHH medulloblastoma patients to inform the next generation of trials.

### 7.3. Imaging and Radiotherapy-Related Biomarkers

Image analysis driven by deep learning enables new insights into tumor biology and how it is reflected in the imaging phenotype [83,84,85]. Within this work package, we will combine information derived from imaging data with clinical and genomic data from the other work packages towards two overarching goals: the first is to investigate the genotype-/imaging relationship in medulloblastoma. For this, we will associate clinical data and genomic data with imaging features through an automated pipeline, yielding non-invasive prediction models of the genotype and clinical variables from imaging data, as we already demonstrated for glioma [86]. We will also correlate—secondly—imaging and radiotherapy with clinical data to deepen the understanding of patterns of treatment failure [87,88] and radiotherapy-associated neurotoxicity. A flexible data analysis platform will be developed that allows integrative analysis of imaging, clinical, and genomic data. Results from this subproject have the potential to assist clinical decision-making both for targeted therapies as well as radiotherapy. In addition, the potential to non-invasively observe genotype changes may help to shed further light on the development of treatment resistance.

In addition, we will also compare proton-based and photon-based radiotherapy in view of their toxicity on craniospinal axis function and on other systems including bone marrow function, neuro-cognitive function, hearing, endocrine function, and fertility.

### 7.4. Neurocognitive Function

In post-pubertal patients with medulloblastoma, information on the mechanism of development of deficits in neurocognitive function (NCF), and thus insight into the potential future avenues for preventing treatment-related neurotoxicity, is widely lacking. NCF impairment after craniospinal irradiation is more severe in young patients with medulloblastoma, but is also prevalent in those irradiated as adults [89]. Impairments negatively affect daily life activities and psychosocial functioning [90]. In addition, NCF not only has independent prognostic significance on survival [91], but neurocognitive deterioration also indicates tumor progression before signs of disease recurrence are evident on MRI [92].

Since a combination of brain lesions, treatment side effects, and psychological distress is likely to contribute to neurocognitive dysfunction in an individually unpredictable way, it is most pragmatic to choose a core testing battery that gauges a broad range of neurocognitive functions. The test battery that meets this prerequisite in the best way has successfully been used in a number of multicenter clinical trials and has been recommended by the International Cognition and Cancer Task Force [93]. This clinical trial battery comprises the Hopkins Verbal Learning Test—Revised (HVLT-R) for total recall, delayed recall, and delayed recognition indexing verbal learning and memory [94]; the Trail Making Test (TMT part A and part B), which measures attention, visual–motor scanning speed, and executive function, and the Controlled Oral Word Association Test (COWA), which evaluates the spontaneous production of words under restricted search conditions [95]. In patients with medulloblastoma, cerebellar compromise due to tumor infiltration or treatment side effects may give rise to distinct deficits in executive functioning, linguistic processing, spatial cognition, and affect regulation. Therefore, NCF assessment in this trial will also include the cerebellar cognitive affective/Schmahmann syndrome scale [96].

The NCF tests will be administered by centrally trained and certified healthcare personnel—for example, a research nurse or neuropsychologist.

### 7.5. Health-Related Quality of Life

HRQoL is a highly relevant domain in the outcome of young adults with cancer [97,98]. Although data are scarce, previous studies have shown that HRQoL outcomes in adult medulloblastoma patients are poor directly postoperative, but improved during treatment up to 30 months post-treatment, after which scores deteriorate again [21,99]. In the 1634-BTG study, HRQoL outcomes will be assessed longitudinally to determine the impact of the tumor and different treatment regimens on aspects of HRQoL and survivorship during the disease course. We will correlate health-related quality of life (HR-QoL) and neurocognitive function (NCF) data at baseline and during the treatment course.

The primary HR-QoL scale that is considered relevant for this study is social functioning, as patients are typically younger adults with an active family and social life. The other scales from the EORTC QLQ-C30 and QLQ-BN20 [100] questionnaires will be considered exploratory in nature. Selected items from the survivorship questionnaire QLQ-SURV111 will also be used [101], including issues related to recurrence, mortgage/loans/insurance, job opportunities and life plans/goals, and relationships with friends and family. Mean HRQoL and survivorship scores over time, as well as mean changes in scores over time, will be evaluated. Relevant subgroup analyses (e.g., SHH subgroup vs. WNT subgroup vs. group 3 and group 4; or proton- vs. photon-based craniospinal radiotherapy) will be performed and HRQoL outcomes will be compared with data from the pediatric population in order to determine similarities in neurotoxicity.

### 7.6. Fertility and Endocrine Function

Radiotherapy and systemic therapy in medulloblastoma patients have a high risk of causing loss or damage of gonadal tissue or gametes or loss or reduction in sex hormones. These abnormalities may result in reproductive complications in survivors of medulloblastoma [102,103]. Potential and actual infertility affects the future quality of life of patients, leads to psychological distress, and is a predictor of stress in present and future relationships [104].

With the development of fertility preservation strategies and oncofertility care, which have been laid down in international guidelines, an increasing number of patients of reproductive age are being referred for fertility preservation and may be able to plan for a biological child after cancer treatment [105]. However, there are a number of barriers to delivering oncofertility care [106], including the lack of available reproductive information that is documented in clinical trials’ protocols about the gonadotoxic and teratogenic risk of new treatment modalities and recommendations for oncofertility care [107].

Very limited gonadotoxic data are available for patients with medulloblastoma and patients taking SMO inhibitors [108]. As part of the EORTC 1634-BTG/NOA-23 study, protocol patients will therefore have an opportunity to consent to an additional fertility substudy. This prospective longitudinal fertility study will describe the uptake and utilization of oncofertility care as well as psychosocial and mental health-related issues surrounding cancer patients of reproductive age. Patients will complete a fertility questionnaire at diagnosis and 12 months, 36 months, and 60 months after the end of treatments. In sites suitable for more detailed substudies, female patients will be assessed for menstrual cycles, reproductive hormones, and antral follicle count measured on pelvic ultrasound scan. Male patients will be assessed for testicular volume, semen analysis, and reproductive hormones.

### 7.7. Comparison of Data with Data from Pediatric Trials

Important efficacy, safety, and translational endpoints from clinical data as well as biomaterial and imaging data raised in EORTC 1634-BTG/NOA-23 will be statistically compared to the pediatric and adolescent SIOP-PNET5-MB medulloblastoma trial (ClinicalTrials.gov: NCT02066220, 26 May 2020) [37]. Endpoints of EORTC 1634-BTG/NOA-23 and functional scores used within the study have been harmonized with PNET5.

## 8. Summary and Outlook

EORTC 1634-BTG/NOA-23 will be the first prospective randomized trial in post-pubertal pediatric and adult patients with medulloblastoma worldwide. In view of novel combination therapies, it will, for the first time, use a targeted therapy, sonidegib, in combination with radio-chemotherapy in a randomized setting, based on evaluation of the genetic subtype of medulloblastoma, and will therefore be personalized. Its main objectives will be to investigate if there is increased efficacy in the SHH subgroup due to the addition of the SMO inhibitor, sonidegib, and to assess in the whole population whether a reduction in radiotherapy toxicity can be attained without compromising efficacy by using a lower dose of radiotherapy.

Translational projects including molecular subgrouping, biomarker design, MRI-imaging evaluation, radiotherapy quality assurance, and evaluation of neuro-cognitive function, health-related quality of life, and fertility/endocrine function are closely connected to the trial. As a result of this, EORTC 1634-BTG/NOA-23 will improve the characterization of medulloblastoma in post-pubertal adolescents and adults through clinical, molecular, and imaging biomarkers. EORTC 1634-BTG/NOA-23 will thereby have a potentially high impact on relieving the burden of medulloblastoma in post-pubertal patients. Information gleaned from this study will ultimately help to decrease short- and long-term toxicity and thereby inform therapeutic teams on how to best reintegrate affected patients into their social and professional lives.

Both the EORTC 1634-BTG/NOA-23 trial and the connected translational work packages aim to support research that advances therapeutic approaches while providing the best possible outcome with the least toxicity for each individual patient. EORTC 1634-BTG/NOA-23 will therefore generate a wealth of data that can be explored in view of future clinical-translational and basic science-translational development, not only in patients with medulloblastoma but also in other rare cancers.
